# On the Use of Galvanism in Lumbago, Sprains, and Some Other Painful Affections of the Muscles and Joints

**Published:** 1846-10

**Authors:** M. Raciborski


10. *On the use of Galvanism in Lumbago, Sprains, and some other painful affections of the Muscles and Joints.* By M. Raciborski. (Gazette Medico-Chirurgicale—from Med. Chir. Review. —M. Raciborski observes that the utility of Galvanism in paralysis of particular nerves is well known, and that Magendie has proved by many recent cases its service in neuralgia generally, but especially in that of the branches of the fifth pair. Having witnessed many successful applications of this kind, mostly in the wards of M. Bouillaud, the author was led to believe the employment of galvanism might be advantageously extended to other affections characterized by violent pain and the absence of signs of inflammation, as muscular rheumatism and *lumbago.* His experiments have been highly successful, the suffering of this last painful affection being frequently forthwith relieved, after the patient had long tried other remedies in vain. The same may be observed of *rheumatism* affecting the muscles of the extremities. It is not easy perhaps to state the *modus operandi* of the remedy; but it would seem to be by directly subduing the *pain*, which prevents the contraction of the muscles, that galvanism produces the instantaneous relief seen in some cases. "Certain it is that, in many cases, we have applied galvanism with some success, even to painful swellings of the knees, rendering walking, if not impossible, at least very painful. Certainly galvanism did not cause the swelling to disappear, but the pain became dissipated, or so diminished as to allow the patient to walk about. We do not doubt that the forced contraction which the galvanic shock produces in the fibres of the muscles, rendered motionless by the rheumatism, must contribute considerably to the good effects derivable from this means."

Four or five cases are given which were relieved almost immediately by galvanism, or rather, perhaps, we should call it galvanic acupuncture, inasmuch as needles were inserted in the parts where pain prevailed, and then brought in contact with the galvanic battery. A very few shocks, which usually themselves caused considerable temporary pain, sufficed to give relief, and enable the patient to exert muscular action without suffering. One or two of the cases seem to us, however, to have all the characteristics of hysteria—but this matters little, inasmuch as an effectual means of relieving the pain of that troublesome affection is a *desideratum.*

"Since our notes were taken, we have had other opportunities of applying galvanism in analogous cases, and always with the same success; but at present we merely desire to draw the attention of practitioners to this new mode of treatment, we need not extend the paper by citing the particulars. Nevertheless, we cannot terminate it without signalizing the admirable effects which galvanism produces in the treatment of *Sprains.* Every one knows that a sprain, although apparently a slight affection, often exacts much time for its cure. When it implicates the ankle or knee, it is not uncommon to see patients deprived of the use of their limbs during two or three months. It is the violent pain felt upon the slightest movement of the part (we are speaking only of simple, uncomplicated sprain,) which retards the cure. The other symptoms are of little consequence, and are usually dissipated promptly. Now, just as we have seen in lumbago, so in sprain, galvanism relieves this pain instantly, and allows the patient to walk without lameness."

M. Raciborski suggests that the galvanism may act by restoring the contraction and tension of the fibres of the articular capsule, (and perhaps those of the tendons,) which had been inordinately distended and elongated by the accident.—*Southern Medical Journal.*

